# Natural Phenol Polymers: Recent Advances in Food and Health Applications

**DOI:** 10.3390/antiox6020030

**Published:** 2017-04-14

**Authors:** Lucia Panzella, Alessandra Napolitano

**Affiliations:** Department of Chemical Sciences, University of Naples “Federico II”, Via Cintia 4, Naples I-80126, Italy; panzella@unina.it

**Keywords:** tannins, lignins, grape pomace, spent coffee grounds, bioinspired phenolic polymers, black foods, food supplement, animal feed, food packaging, tissue engineering

## Abstract

Natural phenol polymers are widely represented in nature and include a variety of classes including tannins and lignins as the most prominent. Largely consumed foods are rich sources of phenol polymers, notably black foods traditionally used in East Asia, but other non-edible, easily accessible sources, e.g., seaweeds and wood, have been considered with increasing interest together with waste materials from agro-based industries, primarily grape pomace and other byproducts of fruit and coffee processing. Not in all cases were the main structural components of these materials identified because of their highly heterogeneous nature. The great beneficial effects of natural phenol-based polymers on human health and their potential in improving the quality of food were largely explored, and this review critically addresses the most interesting and innovative reports in the field of nutrition and biomedicine that have appeared in the last five years. Several in vivo human and animal trials supported the proposed use of these materials as food supplements and for amelioration of the health and production of livestock. Biocompatible and stable functional polymers prepared by peroxidase-catalyzed polymerization of natural phenols, as well as natural phenol polymers were exploited as conventional and green plastic additives in smart packaging and food-spoilage prevention applications. The potential of natural phenol polymers in regenerative biomedicine as additives of biomaterials to promote growth and differentiation of osteoblasts is also discussed.

## 1. Introduction

Investigation of biomass as low cost sources of sustainable chemicals for direct exploitation or further to suitable transformations has become a main focus of academic and applied research over the last few years.

Most of the vegetable sources currently investigated are rich in polymeric phenolic components that are amenable for several uses and applications. In the following part of this review, we will focus on selected classes of phenolic polymers with particular attention to those that can be obtained from abundant available sources by minimal processing.

A considerable number of reports described the uses of such materials in different fields ranging from bioremediation [1] to material sciences, e.g., for preparation of phenolic resin, polyurethane, flocculants, gel, modified polymer materials or adhesives [2]. Apart from this, the great beneficial effects of natural phenol-based polymers on human health and their potential in improving the quality of food have been increasingly appreciated, and this work will critically review the most interesting and innovative reports in the field of nutrition and biomedicine that have appeared in the last five years. Patents were excluded since the main aim of this review is an update of those applications that have a potential for further development, but may not be ready for a straightforward use in industries.

After a short presentation of the main sources of the natural phenol polymers either dietary or not, with special emphasis on waste materials from agro-based industries, polymers obtained through biomimetic procedures from naturally-occurring phenol compounds will be briefly surveyed. The main body of the review will address the applications of phenol-based polymers of relevance to food and health.

## 2. Main Sources of Natural Phenol Polymers

### 2.1. Dietary Sources

Many largely consumed foods are rich sources of phenol polymers, most commonly tannins, traditionally categorized into the hydrolyzable and the condensed or non-hydrolyzable classes [3].

**Figure 1:**
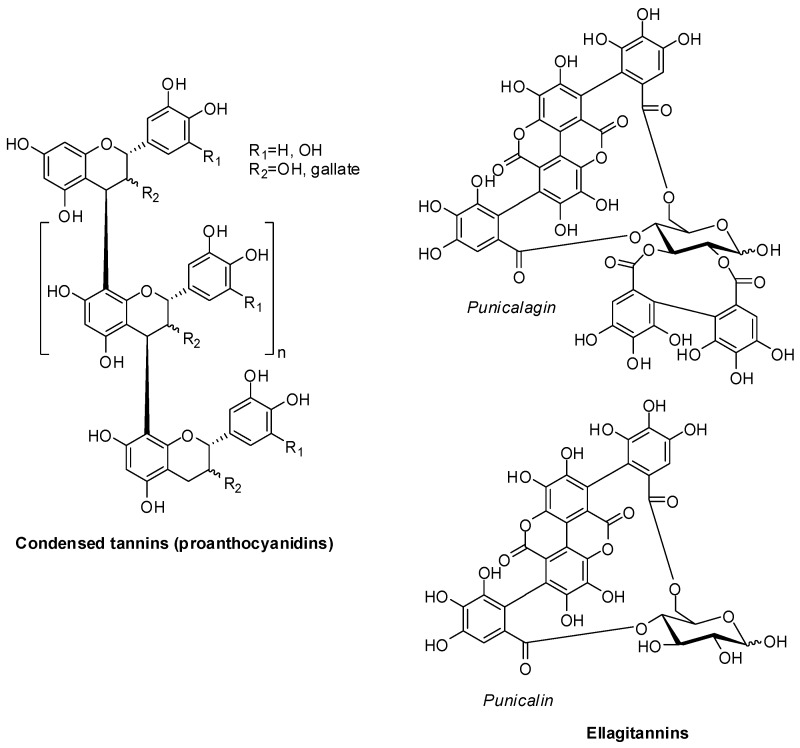
Structure of the main tannins found in grape, persimmon and pomegranate.

One of the most investigated are those found in grape skin and seeds, mainly polymeric proanthocyanidins belonging to the group of condensed tannins that can release monomeric phenols only upon depolymerization under acidic conditions (Figure 1). The distribution and levels of these tannins vary significantly as a function of the grape variety and the degree of ripening and markedly affect the taste and the astringency of the wine produced [4,5,6,7,8]. Of the same type are tannins occurring in persimmon, a fruit largely consumed in Japan [9].

Pomegranates are one of main highly valuable sources of ellagitannins that are also found in other fruits and nuts, e.g., strawberries, raspberries, blackberries, cloudberries, muscadine grapes, almonds and walnuts [10]. These tannins were formulated as hydrolyzable conjugates containing one or more hexahydroxydiphenoyl groups that esterify a sugar, usually glucose. The unique gallagyl esters, punicalagin and punicalin, are the predominant ellagitannins of pomegranate (Figure 1). They mainly occur in the husk (pericarp) and peels (mesocarp) and are extracted into the juice upon commercial processing of the whole fruits. Analyses and quantitation of ellagitannins are particularly difficult because they are only partially solubilized in the extraction solvent or remain covalently bound to cell walls and other macromolecules of the fruit [11].

**Figure 2:**
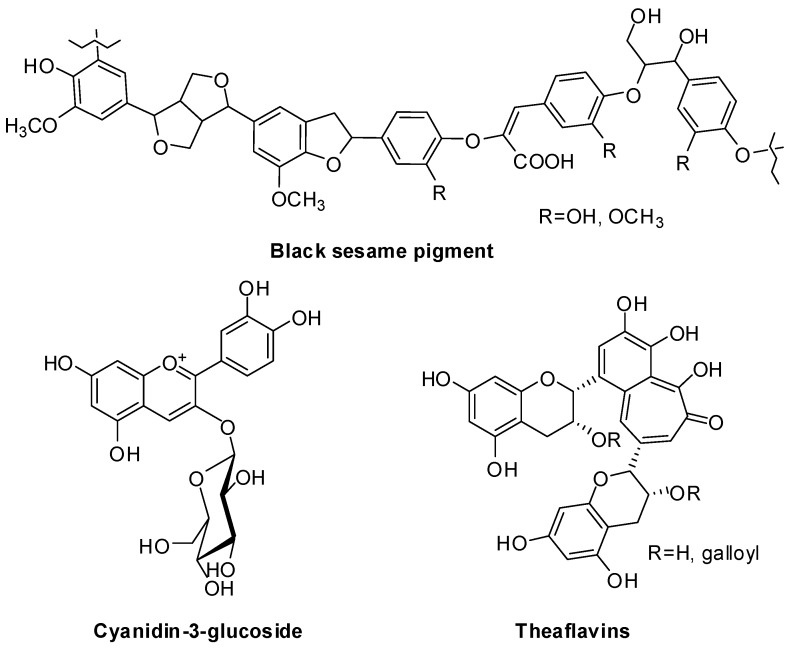
Main determinants of black color in food.

The current burst of interest in the black components of various foods or drinks of plant origin, including rice, tea, beans, radish, ginseng and grapes, stems from the beneficial health properties attributed to the pigments as evaluated in vitro and in vivo studies by comparison with the colorless varieties [12,13]. Actually, structural studies showed that, though all appearing black, the pigments are very different in nature. Paradigmatic is the case of black sesame seeds (*Sesamum indicum* L.), traditionally used in Chinese folk medicine and as food in China and other East Asian countries and largely investigated for its potent antioxidant activity that is considered superior to that of white sesame seeds [14,15]. The black pigment was purified by hydrolytic treatment of the seeds after removal of lipid components, but its insolubility hampered a detailed structural analysis. Solid state spectroscopy coupled to chemical degradation methodologies and model studies indicated coniferyl alcohol-derived units as the main structural components [16,17] (Figure 2).

On the other hand, in many other cases, such as black rice, black beans, sunflower and grapes, structural formulations indicated anthocyanins as mainly responsible for the color observed, whereas catechin polymers were identified as the main components of black tea pigments [18,19,20,21,22,23,24] (Figure 2).

### 2.2. Non-Edible Sources

**Figure 3:**
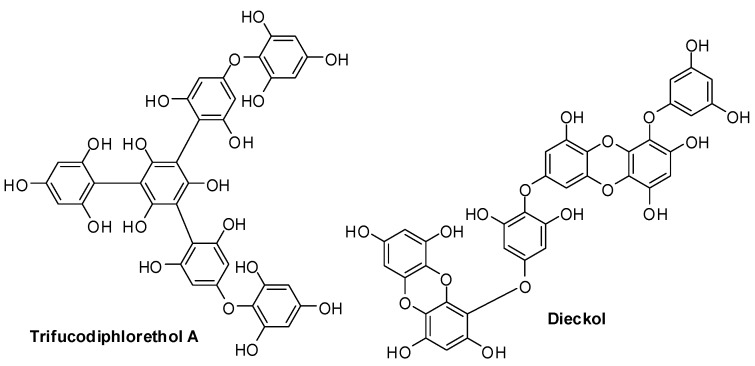
Examples of phlorotannins.

Phlorotannins are a peculiar class of tannins that are produced exclusively by marine brown seaweeds in response to different external stimuli and take part in cell wall structure [25] (Figure 3). A variety of brown algae were investigated as sources of this family of polyphloroglucinols, e.g., *Sargassum muticum* that was shown to contain mainly fuhalols, hydroxyfuhalols and phlorethols, while in other varieties, like *Fucus serratus*, *Fucus vesiculosus*, *Himanthalia elongata* and *Cystoseira nodicaulis*, low molecular weight phlorotannins corresponding to 4–12 monomers of phloroglucinol with different extents of positional isomerism were identified as the main components [26,27,28].

**Figure 4:**
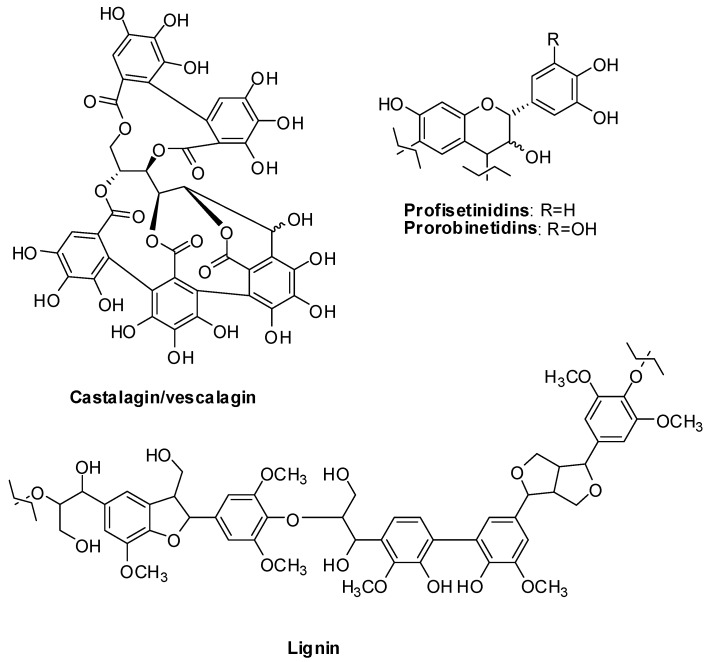
Representative structures of the main phenolic polymers present in wood.

Wood has traditionally represented a rich source of biopolymers. Tannins of tropical woods are typically of the catechol type, whereas those present in temperate woods are of the gallic type. Most investigated are oak tannins that can be easily extracted by repeated treatment with hot water and are composed mainly of hydrolyzable gallotannins including grandinin/roburin E, castalagin/vescalagin, gallic acid, valoneic acid bilactone, monogalloyl glucose, digalloyl glucose, trigalloyl glucose, ellagic acid rhamnose, quercitrin and ellagic acid [29] (Figure 4). Gallotannins including vescalagin and castalagin represent the major constituents also of chestnut tannins [30].

Condensed tannins are commonly extracted from other wood varieties including pine (*Pinus radiata*), mimosa (*Acacia mearnsii*) and quebracho (*Schinopsis lorenzii*). MALDI-TOF analysis indicated that mimosa tannins are predominantly composed of the highly branched prorobinetidins, while quebracho comprises mainly profisetinidins that are mainly linear (Figure 4). These structural features are responsible for the observed differences in the viscosity properties of water solutions and the reactivity to hydrolytic treatment [31].

Several methodologies were developed also for the extraction of lignin from woody biomass, its depolymerization and in some cases further manipulation. Valorization of lignins to get fine chemicals [32] or bio-oil [33] relies on fine tuning of each of these steps, which may range from bioengineering to get lignins featuring a restricted subset of linkages or precursor units in order to facilitate chemical (or enzymatic) deconstruction [34], to a catalytic treatment to get a specific chemical transformation or cleavage of a specific linkage [35], to the delignification or removal of lignocellulosic matrix via lignin depolymerization that is commonly performed via Na_2_S/NaOH treatment yielding the kraft lignin [36,37] or to organic solvent extraction [38].

The most advanced analytical methodologies allowed the identification and structural characterization of low molecular weight species, e.g., the lignin *p*-bis(2,6-dimethoxyphenol)yl dimers [39], but also characterization of the differences between native lignins [40] and technical lignins resulting from the above-mentioned processing [41,42,43,44,45,46] (Figure 4).

### 2.3. Wastes

The biorefinery approach, that is the production of chemicals by agro-waste processing, is nowadays largely pursued to get low cost precursors of value-added materials.

Grape marc, also known as grape pomace, is one of the most investigated bio-wastes derived mainly from grape skin and seeds, which is produced as a byproduct of winemaking on the million-ton scale annually. Presently, the most important high-value use of grape pomace is in the production of oenological tannins, widely-used additives in the food and beverage industry, but methodologies for the extraction of other components like thiol compounds [47,48], monomeric phenols, gallic acid, flavan-3-ols, flavonoids, stilbene and anthocyanins [47,49], as well as specific fractions of condensed tannins [50] were developed. Analytical methods to quantify the tannins, monomeric and oligomeric flavan-3-ols [51], as well as to evaluate the optimal extraction conditions [52,53] were implemented.

Though so far less exploited, byproducts of pomegranate processing for juice production, including peel, pith, carpellary membranes and seeds are gaining interest as a convenient source for extraction of ellagitannins or also phenol monomers following hydrolytic processes [54,55].

Spent coffee grounds are the main residue of the coffee industry, with a worldwide annual generation of six million tons. Several methodologies were developed for valorization of spent coffee grounds by recovery of the polysaccharide fraction, lipids and low molecular weight compounds like chlorogenic acid and other monomeric phenolics or caffeine [56,57,58,59,60,61], or for the production of biodiesel [62,63,64,65].

Hydrolytic treatment proved useful for improving the antioxidant power of spent coffee grounds. Solid state carbon nuclear magnetic resonance (NMR) and infrared spectroscopy in the attenuated total reflectance (ATR) mode indicated that the treatment results mainly in removal of the cellulose/hemicellulose components and provided evidence for the presence of tannins and lignins as the main phenolic polymeric components as supported also by the intense electron paramagnetic resonance (EPR) signal characteristic of carbon-centered radicals [66].

Nutshells have so far mainly been regarded as an alternative source of energy through pyrolysis or gasification processes [67,68]. The most investigated are those associated with nuts largely used for extraction of oil like palm or argan. Furthermore cashew shells have received some attention for the presence of high levels of the long chain phenol cardanol, which can replace phenol itself and is used for the preparation of a variety of resins or other materials [69], while pecan nutshells were shown to contain high contents of condensed tannins [70,71].

### 2.4. Phenolic Polymers by Oxidation of Natural Phenols

**Figure 5:**
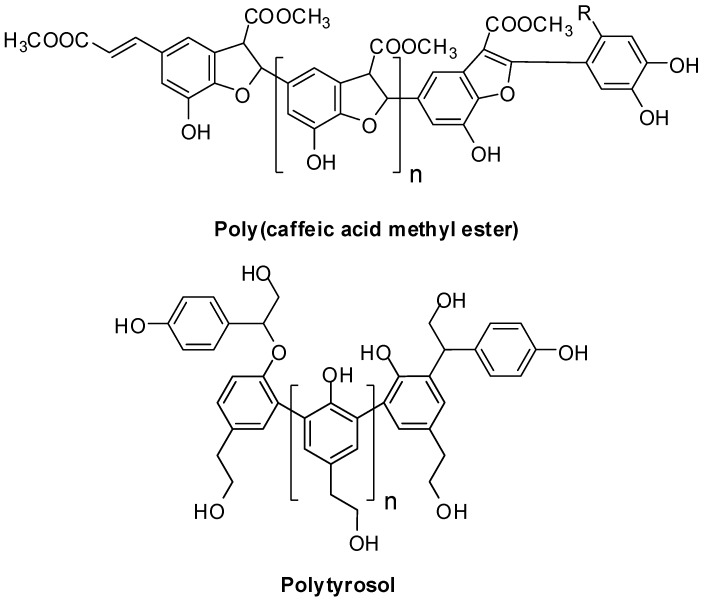
Proposed structures for some bioinspired phenolic polymers [79,82].

Peroxidase-catalyzed polymerization of natural phenols is attracting growing interest as a means to produce biocompatible and stable functional polymers under mild and eco-friendly reaction conditions [72,73,74]. The reaction on monophenolic substrates proceeds via one-electron oxidation leading to the generation of phenoxyl radicals intermediates, which evolve via sequential oxidative coupling steps [75]. Horseradish and soybean peroxidase were used for polymerization of *m*-cresol [76], cardanol [77] and guaiacol [78]. Other relevant examples of bioinspired phenolic polymers include poly(caffeic acid methyl ester) [79], poly(pyrogallic acid) [80,81] and polytyrosol [82] (Figure 5).

## 3. Main Applications of Phenol-Based Polymers in Food and Health Research

### 3.1. Food Supplements and Functional Foods

In this section, we will review recent experimental works aimed at assessing the beneficial properties of a given plant source or of bioactive enriched preparations either by in vitro assays or by trials on animal models and humans. Based on the observed bioactivities or the health benefits, most of these studies propose the use of these plant materials as a food supplement, or prospect the design of a functional food or feed, or ultimately the formulation of a nutraceutical.

Given the massive body of literature on this subject, to adhere to the main focus of this review on phenolic polymers, we chose to rank the different studies on the basis of the nature of the main components of the plant source under investigation.

#### 3.1.1. Tannins

Antimicrobial activity, one of the most established property of tannins, is proposed for the control of the main spoilage bacteria of food in the perspective of implementing antimicrobial food packaging [83]. Persimmon tannins showed an effective activity against a broad range of pathogenic viruses [84], while a more general antioxidant activity was associated with gallotannins of *Ampelopsis grossedentata* leaves tea (Tengcha) [85] and red grape pomace, whose effects persisted on storage and gastrointestinal digestion [86]. The mechanisms responsible for these effects were identified in the increase of the expression of critical antioxidant enzymes like catalase, superoxide dismutase 1, heme oxygenase 1 and gamma-glutamylcysteine synthetase in muscle and endothelial cells [87]. An extract with anti-inflammatory properties on N13 microglia cells stimulated with lipopolysaccharide was obtained by protease digestion of grape pomace [88].

The ability of tannins to bind proteins and particularly proline-rich proteins that prevail in gluten was considered as a therapeutic approach toward celiac disease. In vitro studies showed the ability of procyanidin B3, procyanidin trimers, procyanidin tetramers and an oligomeric mixture of high molecular weight procyanidins to bind to wheat gliadins [89].

A number of in vivo studies has demonstrated the effective activities of tannins in the control of metabolic diseases indicating this class of polyphenols as promising food supplements. Of particular interest are high molecular weight tannins from persimmon fruit that were shown to exert hypolipidemic effects on high-cholesterol diet fed rats through stimulation of serum lecithin cholesterol acyl transferase activity [90]. They were also shown to control the metabolism of lipid and glucose in type 2 diabetes, via induction of uncoupling protein-1 and -3 in brown adipose tissue as shown in a trial using type 2 diabetic mice [91]. Similar effects were evidenced for grape tannins [92]. The antidiabetic action was also claimed for Cabernet Franc Grape pomace aqueous extracts that proved able to affect the levels of circulating peptide hormones related to glucose homeostasis, including glucagon-like peptide 1, glucagon, dipeptidyl peptidase-4 and insulin in mice with high fat diet-induced diabetes [93]. In similar animal models, supplementation with grape pomace or aqueous extracts counteracted adiposity, inflammation, liver damage and impaired insulin signaling associated with metabolic syndrome [94]. Hypocholesterolemic and anti-obesity effects of grape seed flours from white and red winemaking processing were described on high-fat fed hamsters [95].

A moderate consumption of grape pomace extract was proposed for the prevention of colitis development following the observed favorable effects on rats with dextran sulfate sodium-induced colitis [96]. Among the other beneficial effects of grape pomace is vasoprotection, which was attributed to the presence of catechin, but also of additional components liberated during the enzymatic extraction process and accounted for in terms of protection against the deleterious vascular effects evoked by reduced nicotinamide adenine dinucleotide phosphate (NADPH) oxidase activation and superoxide dismutase inhibition [97].

Not many are the reports on formulations for the oral delivery of tannins. Kafirin microparticles encapsulating sorghum condensed tannins were proposed as a nutraceutical to attenuate hyperglycemia and control type 2 diabetes [98].

The health beneficial effects of tannins were widely explored also on productive livestock, but the results are sometimes inconclusive or controversial. Most investigated are condensed tannins, primarily quebracho tannins. Milk fatty acid profile was evaluated in German Holstein dairy cows receiving a basal diet integrated with quebracho tannin extracts, with particular reference to odd/branched fatty acids as biomarkers of the rumen microbial protein synthesis. Positive effects on milk protein content were observed only at moderate intakes of quebracho [99] or acacia [100] tannin extracts, whereas in other cases, the results were not conclusive of a positive effect [101]. In dairy ewes, quebracho extracts produced effects on fatty acid milk profile and microbial species more marked than those observed with hydrolyzable chestnut tannins [102,103].

Supplying either condensed (acacia and quebracho) or hydrolyzable (chestnut or valonea) tannins had the potential to reduce methane production and ruminal protein degradation with minimum detrimental effects on the efficiency of ruminal fermentation [104,105,106]. Effects of ingestion of quebracho or other non-hydrolyzable tannins on different processes were described, e.g., levels of salivation [107], anthelmintic effects [108,109,110,111,112] and improvement of meat quality [113].

Profisetinidin tannins from quebracho induced antioxidant effects in animal tissues without being degraded or absorbed in the gastrointestinal tract [114]. Similarly, chestnut tannins improved the antioxidant status of heat-stressed lambs [115] or the fatty acid composition of eggs [116] and reduced intestinal skatole production in pigs [117,118].

Grape pomace tannins were also tested as feed supplements, though to a minor extent compared to wood tannins. Grape pomace tannins supplementation was reported to: (i) improve in vitro true digestibility, rumen fermentation end-products, as well as to reduce methane production on swamp buffaloes [119]; (ii) decrease oxidative stress-induced damage to lipids and proteins and enhance growth of facultative probiotic bacteria while inhibiting growth of pathogen populations, such as *Enterobacteriaceae* and *Escherichia coli* in lambs [120]; (iii) decrease the levels of fatty acid peroxidation in plasma and egg yolk [121]; whereas other studies reported no statistically-significant effects [122].

**Figure 6:**
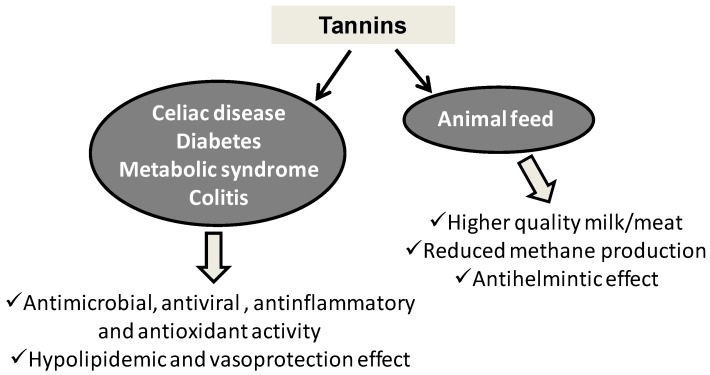
Main applications and effects of tannins as food supplements.

An overview of the main applications and effects of tannins as food supplements is shown in Figure 6.

#### 3.1.2. Lignins

Lignins were far less investigated either as supplements for animal feeding [123] or in in vitro assays. Lignosulfonic acid produced from the sulfite pulping of softwood was able to inhibit α-glucosidase activity and intestinal glucose absorption in colorectal adenocarcinoma cells suggesting that it can serve to control the rise in blood glucose levels [124]. Oil palm empty fruit bunch lignins, extracted with three types of techniques (soda, kraft and organosolv) comprising mainly *p*-hydroxyphenyl, guaiacyl and syringyl units exhibited a marked antioxidant activity on in vitro assays and inhibited glutathione-S-transferase enzymes activity in rat liver cytosolic fractions [125].

#### 3.1.3. Black Foods

This group embraces a variety of dark brown, deep violet and black pigmented foods with a potential as dietary supplements. Many of these were traditionally consumed in East Asia, but their beneficial properties have been appreciated only recently following a considerable number of in vitro studies and trials on model animals and humans. Though the structural features of the pigments characterizing these foods are indeed very different or even not yet defined, they are presented altogether using the plant source as the sole criterion for ranking.

The activity of black soybean in the control of obesity and other metabolic diseases has long been documented. Black soybean koji derived from *Aspergillus awamori* fermentation was shown to improve glucose uptake by modulating glucose transporters 1 and 4 expression in a 3T3-L1 insulin-resistance adipocytes cells, suppressing differentiation and lipid accumulation [126]. Related effects on hyperglycemia and insulin sensitivity were observed in diabetic mice [127]. A similar activity was reported for black adzuki bean (*Vigna angularis*) extracts [128]. The broths from fermentation of black soybean in the presence of specific ingredients exhibited high antioxidant potency on account of the high levels of polyphenols including flavonoids and anthocyanins together with essential amino acids, which makes this material of interest for the preparation of nutritional drinks and health foods [129]. The anti-obesity, blood glucose control, cholesterol and insulin resistance lowering effects of black soybean either fermented or not were confirmed on animal models including hypercholesterolemic rabbits [130] and mice with high fat/cholesterol diet-induced obesity [131,132]; in addition, in ovariectomized mice, fermented black soybean supplementation lowered serum HDL-cholesterol, estradiol levels, serum alkaline phosphatase activity and osteocalcin levels, suggesting an osteoporosis preventive action [133].

The health beneficial effects of black tea, one of most consumed beverages in the world, are a common belief, but only recent studies provided evidence for specific activities. Amelioration of oxidative stress and modulation of the antioxidative system were demonstrated in a rat model of quinocetone-induced renal dysfunction [134] or rats with HgCl_2_-induced oxidative damage [135]; moreover, experiments with ovariectomized rats indicated black tea extracts as a suitable alternative for calcium supplementation in the early stage of menopausal osteoporosis [136]. An issue that was addressed in human trials is the understanding of short- and long-term effects. Body weight and fat distribution were positively affected by regular consumption of black tea over three months, but these effects were lost on a longer period [137], while long-term benefits were reported for blood pressure control [138], and a decrease of cardiovascular risk factor was evidenced in a prospective randomized clinical trial [139]. In addition, consumption of black tea in association with bread fortified with iron and zinc ions favored iron absorption [140]. The polyphenols obtained by hot compressed water extraction of wastes from commercial production of tea beverages proved rich in catechin monomer/oligomers like theaflavin 3-O-gallate, theaflavin 3′-O-gallate, theaflavin 3,3′-O-gallate, epigallocatechin gallate and epicatechin gallate, with high pancreatic lipase-inhibiting action, suggesting the use of this underutilized resource in dietary supplements and medications, for the prevention and treatment of obesity [141].

**Figure 7:**
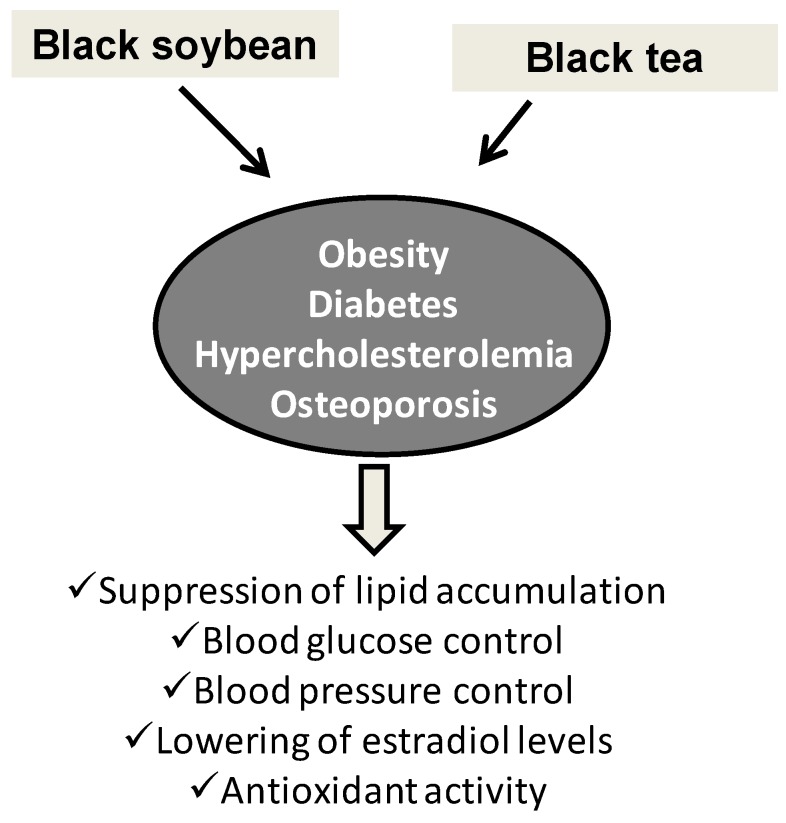
Main applications and effects of black soybean and black tea as food supplements.

An overview of the main applications and effects of black soybean and black tea as food supplements is shown in Figure 7.

The many positive biological effects of black rice were ascribed to the high anthocyanin levels. Support for protective effects against retinal photochemical damage [142], reduction of the risk of hepatic steatosis, hyperlipidemia and hyperglycemia [143,144] and inhibition of angiogenesis and tumor growth [145] were demonstrated in animal trials.

Other black seeds like black sesame [146] or black cumin [147] were used in the preparation of functional foods, particularly bread, to ensure the delivery of the daily intake of zinc, potassium, phosphorous, iron and copper, but also the use for animal feed was considered [148,149]. A marked ability to bind metal ions, particularly toxic heavy metals, was shown for the pigment purified from black sesame seeds [17]. The beneficial effects on lipid profile of dietary supplementation with black seeds, particularly cumin, on diabetic patients were assessed by human and animal trials [150].

Among roots, of particular interest is black ginseng traditionally consumed in Korea and other southeast countries. Anti-obesity effects in a high fat diet supplemented with ethanol extracts of black ginseng were observed on mice models [151].

Similarly to many cruciferous vegetables, black radish was ascribed a positive action on liver by detoxification of drugs and toxins. A pilot trial on healthy male subjects receiving a dietary supplementation of Spanish black radish indicated that this effect is due to induction of phase I and phase II enzymes [152].

### 3.2. Active Packaging and Film Stabilization

Oxidative deterioration of polymers used in food packaging is an issue of considerable economic and practical concern especially under conditions in which loss of quality of the polymer may result in altered performance and limited shelf life. Hindered phenolics, such as butylated hydroxyanisole, butylated hydroxytoluene, *tert*-butylhydroquinone and propyl gallate, are widely used as primary antioxidants for polymers employed in food packaging. Yet, issues regarding possible toxic, mutagenic and carcinogenic effects of these additives have been raised, as previous observations indicated that small phenolic antioxidants may be released from polymer packaging films when in contact with water or beverages [153]. Increasing attention was therefore placed on the use of phenolic polymers as bioavailable, biocompatible antioxidants for polymer stabilization in packaging and related technologies. Expected advantages with respect to the monomers would include lower volatility, greater chemical stability under processing conditions and lower tendency to be released from the polymer into the contact medium (food, water, etc.).

Phenol polymers obtained by oxidation of monomers like guaiacol [78], pyrogallol [80,81], 4-methoxyphenol [154] or methyl caffeate [79] typically using peroxidase are able to increase the oxidation induction time of polypropylene or polyethylene under biomimetic conditions.

Natural phenolic polymers, primarily lignins from wood processing or from lignocellulose wastes, were recently widely explored to confer desired characteristics to traditional or biodegradable polymers used for packaging. Improved properties, e.g., higher viscoelastic and tensile characteristics, reduced oxygen permeability and increased UV resistance, of biodegradable plastics, like poly(3-hydroxybutyrate-co-3-hydroxyvalerate) [155,156], polylactic acid [157] and alginate [158], have been obtained following their processing with kraft lignins or composites fabrication. Furthermore, in the case of polyethylene, blending with kraft lignins improves the thermal stability [159]. Formulations of low cost oxo-biodegradable polyethylene-lignin hybrid composites were prepared starting from ethylene/vinyl acetate copolymer as the compatibilizer and a transition metal salt as the oxo-biodegradation promoter [160]. Films prepared from nanocomposites of lignin and cellulose nanocrystals that combine the oxygen barrier property of cellulose nanocrystal films with the antibacterial property of lignin are also promising materials in food and medical applications [161]. Other agro-wastes rich in antioxidants, such as grape pomace waste, turmeric shavings and waste, spent coffee grounds and orange peel waste were tested as finely-grounded powders without any work-up or purification step as thermo- and photo-oxidative stabilizers for low density polyethylene [66,162]. Similarly, condensed tannin extracts from *Pinus radiata* and *Acacia mearnsii* compounded into linear low-density polyethylene films improved UV stability during accelerated weathering, with greater strength and elongation retention [163].

Waste-derived materials alternative to plastics are actively explored also for the preparation of packaging films, but in most cases, limitations to their full exploitation come from the poor mechanical or other properties that can be greatly improved by incorporation of lignins or tannins in turn derived from waste materials. For example, the poor mechanical and barrier properties of starch-based films obtained from agro-wastes can be improved by incorporating lignin isolated from oil palm black liquor waste as a reinforcing material [164]. Similarly, lignins containing cellulose nanofibrils from oil palm empty fruit bunches were used to reinforce starch-based biofoams as a sustainable and green alternative to polystyrene foams for packaging and insulation materials with comparable mechanical properties [165]. The alcohol-soluble fraction of lignins from durum wheat straw was also incorporated into starch to yield films with high resistance to thermal degradation and good antioxidant activity as a function of the lignin content [166]. Increased protection against UV light as a result of reduced transparency was obtained by inclusion of valonea tannins into films prepared from soya protein isolate [167].

Besides polymer stabilization, development of safe and efficient antioxidants for packaging may be of interest also for the food industry. Oxidative deterioration of packaged food may cause significant decreases in shelf life associated with off-flavors, off-odors, color changes and nutritional loss and is therefore emerging as a major economic and health issue, especially with the ever-increasing search for long life foods. Accordingly, there is an increasing demand on the part of consumers and the market for natural or natural product-derived antioxidants that could be used in the place of synthetic counterparts not only for plastic stabilization, but also as ingredients for active packaging. Furthermore, in this case, the use of natural polymeric phenols as additives of packaging films either from biodegradable or non-biodegradable polymers warrants lower tendency to leaking.

Grass lignins recovered from alkaline industrial processes and from different ethanol organosolvent pretreatment exhibited good antioxidant power in common in vitro tests, which was maintained also for the material embedded in cellulose films [168]. The most active components were shown to be the low-molar-mass phenolics, namely *p*-hydroxycinnamic acids and lignin depolymerization products, with an activity of control of lipid peroxidation comparable to that of rosmarinic acid, a low molecular weight antioxidant most commonly used in food packaging. This observation was also confirmed for poly(lactide)-lignin films in which the low molecular weight fraction was increased by heating in the thermo-compression step of the material processing [169].

Furthermore, grape tannins have been investigated as antioxidant ingredients for the preparation of active packaging films. Incorporation by melt blending into polyolefins [170] or ethyl cellulose [171] films conferred an antioxidant activity in a dose-dependent manner, without altering the original mechanical properties of the materials.

Food packaging with bioactive compounds is an innovative approach also for the prevention of the growth of food-spoilage microorganisms. Antimicrobial assays revealed a capacity to inhibit the bacterial growth of Gram-negative *Erwinia carotovora* subsp. *carotovora* and *Xanthomonas arboricola* pv. *pruni* for polymeric films based on polyvinyl alcohol, chitosan and lignin nanoparticles, suggesting innovative opportunities against bacterial plant/fruit pathogens in food packaging applications [172]. Limes coated with xanthan gum solution containing lignin extracted from sugarcane bagasse showed high antifungal activity [173]. Soluble dietary fiber-tannin self-assembled films exhibited a broad spectrum of antimicrobial properties and excellent cell biocompatibility, which might open up new applications in the food preservation field [174].

Active extracts from agro-industrial subproducts, such as almond shells and grape pomace incorporated into sodium alginate films, exhibited antimicrobial properties against five food pathogenic bacteria, namely *Escherichia coli*, *Pseudomonas aeruginosa*, *Listeria monocytogenes*, *Staphylococcus aureus* and *Salmonella* spp. [175]. Shelf life of durian paste product wrapped with areca palm leaf sheath treated with spent coffee ground extracts was extended from three days to 21 days under the storage condition of 30 °C [176].

**Figure 8:**
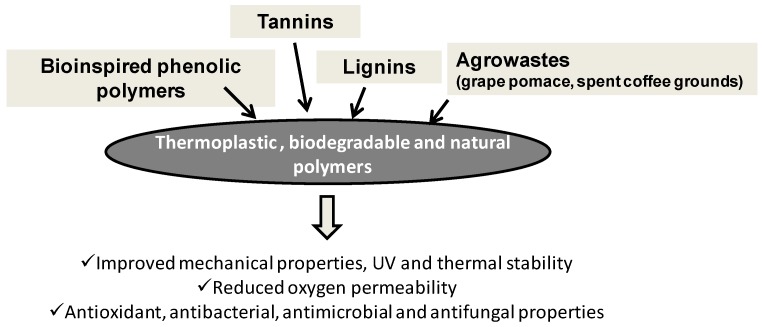
Main effects of natural and bioinspired phenolic polymers for packaging applications.

An overview of phenol polymer application in active packaging and film stabilization is presented in Figure 8.

### 3.3. Tissue Engineering

The possibility to engineer cell microenvironments by means of bioactive materials combined with physical and biochemical stimuli is the basis of regenerative medicine, an innovative approach that overcomes the traditional transplantation or replacement therapies.

Several phenolic polymers were evaluated as biomaterial additives to favor cell growth and differentiation. Thanks to their antioxidant and antimicrobic properties, polyphenols were extensively investigated as promoters of osteoblast differentiation, since they can counteract the inhibitory effects of reactive oxygen species (ROS) on the process of bone formation by osteoblastic cells. Brown algae phlorotannins, such as triphlorethol A, eckol and dieckol, proved effective in enhancing the osteoblast differentiation as indicated by increased alkaline phosphatase activity along with raised levels of osteoblastogenesis indicators and intracellular calcification [177]. Similarly, polytyrosol, a mixture of oligomers from enzymatic tyrosol oxidation, stimulated alkaline phosphatase activity of osteosarcoma SaOS-2 cells at Day 7 also when loaded at 5% *w*/*w* into highly porous polylactic acid scaffolds featuring hierarchical structures, while tyrosol was completely inactive [82].

Hydrogels offer several advantages as biomaterials for bone regeneration, including ease of incorporation of soluble substances, such as mineralization-promoting enzymes and antibacterial agents. When included in gellan gum hydrogels in the presence of alkaline phosphatase, seanol, a seaweed extract rich in phlorotannins, induced mineralization with calcium phosphate uptake and produced an antibacterial activity [178]. Alginate-lignin aerogels that proved non-cytotoxic and with good cell adhesion properties are also attractive candidates for applications in this field [179].

A biomedical scaffold should have a three-dimensional shape that is structurally and mechanically equivalent to the replaced tissue and should provide attractive sites for the injected cells to attach, proliferate and differentiate. Biocomposite scaffolds based on polycaprolactone supplemented with hydrophilic collagen extracted from fish skin and phlorotannins from brown algae exhibited marked calcium deposition and osteogenesis ability compared to the materials not including the polyphenols [180]. Micro-/nano-fibrous substrates were also widely used for tissue regeneration because of their similarities to extracellular matrix components and their high surface area, which facilitates cell attachment and proliferation. Examples include electrospun polycaprolactone-phlorotannin micro/nanofibers that favored osteoblast-like cells (MG63) growth, with higher alkaline phosphatase activity, and calcium deposition with respect to pure polycaprolactone material [181], but even better results in terms of initial cell attachment, cell viability, alkaline phosphatase activity and mineralization were reported for multilayered composite scaffolds consisting of polycaprolactone, β-tricalcium phosphate (β-TCP) and phlorotannins fabricated by a combined melt-plotting and coating system that ensured a specific 3D architecture. A specific role of phlorotannins in these materials is the increase of the wettability of the surface [182]. Biodegradable polylactic acid-lignin composites are considered to be promising renewable plastic materials; the rationale of the addition of lignins to polylactic acid is counteracting the oxidative stress induced by polylactic acid as a biomaterial. Nanofibrous composites were prepared by electrospinning of polylactic acid-lignin copolymers and further blended with poly(l-lactide). These nanofibers, which exhibited excellent radical scavenging capacity, induced higher cell proliferation compared to neat nanofibers on three different types of cells (PC12, human dermal fibroblasts and human mesenchymal stem cells) [183]. Along the same lines, cell culture studies suggested that polycaprolactone/lignin-poly(methyl methacrylate) nanofibers were biocompatible and promoted the proliferation, attachment and interactions of human dermal fibroblasts [184]. The structural properties like porosity, pore size and tortuosity, as well as the surface characteristics are critical parameters of the scaffolds for cell attachment. Hierarchical surface structure obtained on polycaprolactone film by an electrical field-aided nano-/micro-casting technique to mimic a typical natural hierarchical structure, the lotus leaf, coated with tannins to improve the hydrophilicity, induced high cell viability compared to that of the smooth surface, with an increase also in the levels of calcium deposition [185].

**Figure 9:**
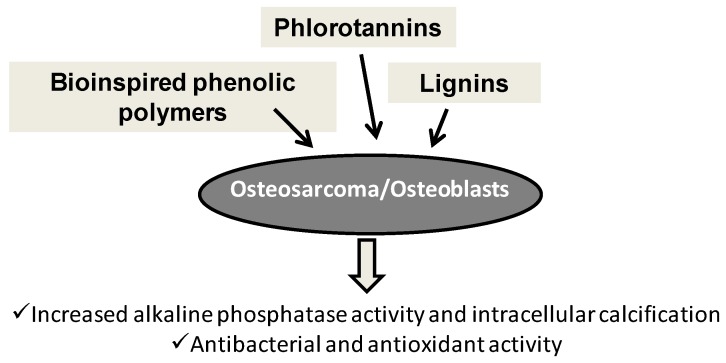
Main effects of natural and bioinspired phenolic polymers in bone tissue engineering.

An overview of phenol polymer application in bone tissue engineering is depicted in Figure 9.

## 4. Conclusions

The wide distribution of natural phenol polymers in commonly-consumed foods, in non-dietary sources and, most importantly, in agro-wastes has stimulated in the last few years intense research work aimed at documenting the many favorable properties of these materials and developing applications in a variety of fields.

**Figure 10:**
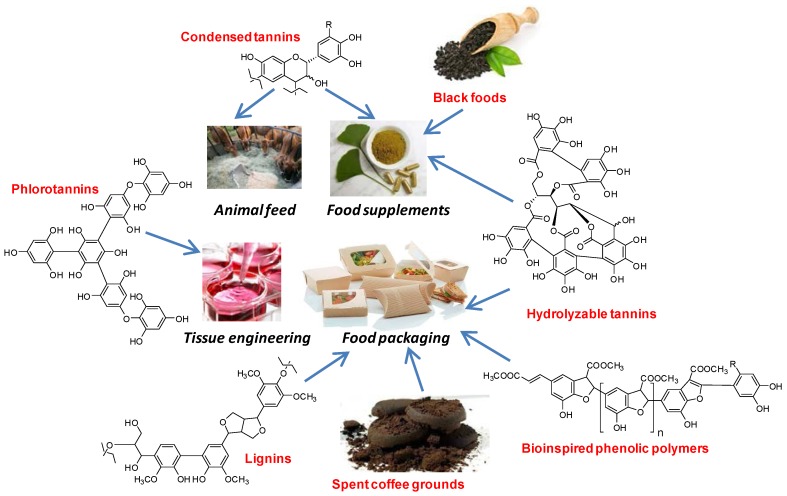
Overview of the main applications of phenolic polymers in food and health research.

Long recognized as antimicrobial agents, tannins have been the focus of continuous interest in the field of animal feeding where a number of trials has demonstrated positive effects particularly of quebracho and other condensed tannins in the well-being of livestock and in the quality of the products thereof (Figure 10).

Though not traditionally regarded as good candidates for food supplements because of the astringency and bitter taste, a number of tannins from different edible sources or food processing wastes, notably persimmon, pomegranate and grape pomace, were shown to exhibit most useful activities in the control of metabolic diseases on animal models. Similar activities of great general interest were documented for a series of black foods that are nowadays greatly appreciated also in Western countries.

Recently discovered, phlorotannins, a class of oligo- and poly-phloroglucinols produced only by seaweeds, were appreciated for their effectiveness in enhancing the osteoblast differentiation and favoring intracellular calcification. It is expected that these tannins will be more and more investigated as additives of biomaterials for tissue engineering purposes.

Food packaging is undoubtedly one the most investigated and innovative fields for the exploitation of phenol polymers. Biocompatible and stable functional polymers prepared by enzyme polymerization of natural phenols proved a valuable alternative to synthetic stabilizers for polyethylene and other conventional plastic films used in food packaging. They offer the additional advantages of a lower volatility, greater chemical stability under processing conditions and lower tendency to be released from the polymer into the contact medium with respect to the monomeric polyphenols that have largely been investigated over the past few years. In addition, also natural phenol polymers mostly derived from wastes comprising grape pomace, turmeric shavings, spent coffee grounds and orange peels, have found application as thermo- and photo-oxidative stabilizers. The use of lignins as a low cost additive capable of ameliorating the poor properties of packaging films obtained from waste materials like starch or cellulose would expectedly support the development of these all-natural packaging materials. Another innovative approach in the field is based on the inclusion of tannins and bioactive extracts from agro-industrial subproducts in biodegradable films for the prevention of the growth of microorganisms responsible for food spoilage.
